# A Transmedia Storytelling Intervention With Interactive Elements to Benefit Latinas’ Mental Health: Feasibility, Acceptability, and Efficacy

**DOI:** 10.2196/mental.8571

**Published:** 2017-10-19

**Authors:** MarySue V Heilemann, Patricia D Soderlund, Priscilla Kehoe, Mary-Lynn Brecht

**Affiliations:** ^1^ School of Nursing University of California, Los Angeles Los Angeles, CA United States; ^2^ School of Nursing University of California, Irvine Irvine, CA United States

**Keywords:** depression, anxiety, transmedia, Internet, mental health, mood disorders, smartphone

## Abstract

**Background:**

Latinos report higher rates of depression and anxiety than US whites but are less likely to receive care. Transmedia storytelling interventions accessible on the Internet via smartphones, tablets, and computers hold promise for reducing reluctance to explore or get help for symptoms because they are private, convenient, and can reach large numbers of people, including Latinas with mental health needs.

**Objective:**

The purpose of this study was to examine the feasibility, acceptability, and preliminary efficacy of a mental health transmedia intervention for Latinas with elevated symptoms of depression, anxiety, or both.

**Methods:**

A total of 28 symptomatic English-speaking Latina women aged 21 to 48 years participated in a 6-week study using a within-group design. All aspects of the study were completed via telephone or Internet. Participants used their personal devices to engage the Web-based transmedia intervention (in English) that included story-based videos, a data-informed psychotherapeutic video, an interactive video sequence, and a blog written from the point of view of one of the characters with links to mental health resources. Perceived confidence to get help and perceived importance for seeking immediate help were both measured using single-item questions. Participants completed surveys at baseline (via telephone) and 1 and 6 weeks after media engagement that measured various factors, including depression (Patient Health Questionnaire; PHQ-9 and PHQ-8) and anxiety (Generalized Anxiety Disorder scale; GAD-7). A telephone interview was conducted within 72 hours of media engagement. Action taken or intentions to get help (single-item question) and talking about the videos with others (single-item question) were measured 1 and 6 weeks after media engagement. Repeated measures analysis of variance was used to assess change in depression (PHQ-8) and anxiety (GAD-7) before transmedia engagement and 1 and 6 weeks after. Spearman correlations evaluated the association of confidence and importance of getting help with action taken, anxiety, and depression.

**Results:**

All 28 Latinas (English speakers) who engaged with the transmedia remained in the 6-week study. Within 1 week of transmedia engagement, 39% of women took action to get help, and 82% discussed the media with others. Symptoms of depression (*F*_2,54_=9.0, *P*<.001) and anxiety (*F*_2,54_=18.7, *P*<.001) significantly reduced across time. Higher levels of confidence were significantly associated with actions taken at 1 (*P*=.005) and 6 weeks (*P*=.04), and higher levels of importance were significantly associated with actions taken at 1 (*P*=.009) and 6 weeks (*P*=.003). Higher levels of confidence were associated with lower levels of depression (*P*=.04) and anxiety (*P*=.01) at 6 weeks.

**Conclusions:**

Preliminary findings indicate a culturally tailored mental health transmedia intervention is a feasible approach that holds promise for engaging large numbers of symptomatic English-speaking Latina women to begin the process of seeking help, as well as decreasing symptoms of anxiety and depression.

## Introduction

### Background

Latinos are the largest ethnic minority group in the United States [1], and they report more symptoms of depression (such as sadness or hopelessness) and more symptoms of anxiety (such as nervousness) compared with US whites [2]. Symptoms of anxiety often co-occur with symptoms of depression [3]. However, US Latinos receive less mental health care than whites [4], even if they have insurance [5,6], and often are not diagnosed despite distress [7]. In a large epidemiologic study, less than one-third of Latinos of Mexican descent with major depression received guideline concordant care [8]. Acculturation processes and social assimilation pressures associated with exposure to life in the United States lead to shifts in language usage from Spanish to English and contribute to intergenerational changes in Latino family stability and cohesiveness, bringing cultural stress that may influence mental health [9]. US-born or English-speaking Latinos report more anxiety and depressive symptoms [9,10], as well as risks for suicidal ideation and suicide attempts [11] compared with foreign-born or Spanish-speaking Latinos. Heilemann and colleagues found that women of Mexican descent aged 21 to 40 years who were born or grew up in the United States reported more depressive symptoms than their immigrant peers [12]. However, talking about a personal mental health problem [13] or receiving mental health treatment can be perceived as stigmatizing in Latino culture [14,15]. Women fear that if they take the step to get mental health care, they will end up being committed to a mental health institution [7] or that other people will think they are crazy. [16]. Despite difficulties in daily life, often only Latinas with the highest levels of symptoms seek professional help [17]. This occurs despite evidence that treatments have been effective in reducing symptoms of depression [18] and anxiety [19] among US Latinas. Barriers to seeking therapy for Latinas include being overwhelmed and feeling responsible to meet loved ones’ needs before seeking resources for their own mental health needs [20]. The daily demands of women’s busy lives as mothers, workers, or daughters [21] raise the need for finding help that is flexible in terms of access, timing, location, and cost. Social and cultural sensitivity of available services is key [22], but depressed ethnic minority women have been found to be less likely to perceive they have a need for mental health care [23]. Becoming aware that depression or anxiety is present and overcoming stigma related to emotional health is crucial to being able to consider using resources such as therapy. For this reason, useful strategies that are also entertaining such as storytelling, hold promise for attracting women to consider their situation and the options for help.

### Storytelling Interventions

Although only able to reach a small audience, an attractive fotonovela booklet created by Cabassa and colleagues [24] was used to help Latina adults overcome stigma for depression. The fotonovela portrayed a story with photos and captions, comic book style, about a fictitious Latina mother who was depressed; review of the booklet led to increased intentions to seek treatment in a sample of at-risk, mostly Spanish-speaking adult Latinas [25]. However, efforts targeting English-speaking Latinos to date are lacking, and the reach of paper interventions with booklets such as fotonovelas is limited. Entertainment education or “edutainment” [26] strategies that involve webnovela [27] and telenovela formats have used compelling characters to showcase a variety of sexual health issues that have influenced Latino audiences since the pioneering work of Miguel Sabido in telenovelas that aired in Mexico in the 1970s [28]. Importantly, a specific focus on mental health for adult Latinas has not yet been approached using the telenovela format as an edutainment strategy. However, telenovelas have been used in health intervention research with US Latinos on topics such as alcohol abuse prevention [29] home care service use for elders with chronic illnesses [30], breast cancer screening [31], prevention of stroke and heart disease [32], colorectal cancer screening [33], and dementia knowledge and attitudes [34].

Transmedia, a form of digital edutainment, moves beyond telenovela viewing and holds promise for innovative interventions because, as a communication technology, it offers a convenient methodology for reaching and affecting large numbers of individuals including those in need. As Latinos have the highest usage of smartphones among US adults (18-49 years) [35] and high Internet usage including both English (94%) and Spanish speakers (86%) [36], they are a prime audience for an innovative approach using transmedia. A compelling media package can be tailored to address mental health–oriented content specifically designed for a Latina audience. Transmedia involves digital storytelling, which is conveyed over multiple platforms [37] including smartphones, tablets, and computers. Content can be delivered via network TV, cable TV, or streaming services (Wi-Fi, Internet, or cellular networks). With transmedia, different parts of a story unfold across various platforms, allowing viewers to make choices about what parts of the story to engage with, how, and when. Thus, stories can become even more captivating as they extend beyond the traditional episodic format through video logs, blogs, websites, individual videos, or a miniseries of short bonus videos featuring characters who not only provide drama and entertainment but who also provide information or resource options. The timing of viewing is up to the viewer, but the higher their interest in the story and characters, the more they are likely to engage [37]. The options for engagement give transmedia an interactive dynamic controlled by the viewer [38,39]. The dynamic features of transmedia contrast with the more static nature of pamphlets, fotonovelas, or comic books and the more passive nature of film or television.

### Transmedia: Storytelling Over Multiple Digital Platforms

Transmedia that includes health messages has become increasingly popular among the growing numbers of English-speaking Latinos in the United States through Wise Entertainment’s hit show, *East Los High* that reached viewers in all 50 US states and 163 countries during its first season [26]. It combined episodic dramas with transmedia extension videos that convey [26] unique parts of the story. Each part contributes to the creation of an immersive media experience [37] that can be leveraged for purposes of health. Whereas *East Los High* has focused on sexual and reproductive health, no transmedia interventions to date have focused specifically on topics related to adult Latinas’ emotional health experiences or dealing with symptoms of depression or anxiety. The demand for story-based media is increasing among US Latinos who, according to Nielsen’s Digital Consumer Report [40], watch more digital media than the average American.

### Goal of This Transmedia Intervention

With the goal of using transmedia in an intervention, we drew upon our previous research data to enhance understanding of key issues for English-speaking Latinas associated with their experiences of depression or anxiety, as well as resilience and coping. We used those deidentified data to create a transmedia intervention called “Catalina: Confronting My Emotions” that was offered to women struggling with mental health symptoms. The purpose of this study was to examine the feasibility of a mental health transmedia intervention for English-speaking Latinas with moderate-to-severe symptoms of depression or anxiety. The first aim was to examine feasibility in terms of implementation, demand, and acceptability of a 6 week “Catalina” transmedia intervention. The second aim was to test the preliminary efficacy of the transmedia intervention to spur change in symptoms, perceived confidence and perceived importance of seeking help, and actions taken to get help.

## Methods

### Development of the Media

With guidance from the University of California, Los Angeles Institutional Review Board (UCLA IRB), we used deidentified data from the Principal Investigator’s (PI) previous mental health studies with English-speaking and second generation Latinas as groundwork for creating composite characters and a storyline. Then, the PI teamed up with a Latino writer-director from Hollywood who created the scripts (in English) for the drama. Content validity for scripts was obtained from a community advisory board of four mental health therapists in two waves of critique, with a focus on making the scripts realistic and culturally appropriate. All four therapists had experience working with English-speaking adult Latinas and two of the four were Latinas themselves. The script was revised after each wave of input from the therapists. Casting was done in Hollywood, Latino or Latina actors were hired, and the video scenes were filmed and edited. Theater testing was done using preliminary versions of the videos, with a community sample of 19 Latina adults. Feedback regarding relatability of the characters and cultural appropriateness of the storyline was collected using focus groups and individual interviews. Feedback led to the final editing of the media. Simultaneously, a computer programmer collaborated with the PI to create an interactive feature and the Web page that was designed to match the look and feel of the videos. It included photos, captions, and links to all the videos and the resource-rich blog, including contact information and links to local and Web-based resources plus hotlines for mental health services.

### Study Design

Approval was granted by the UCLA IRB for a mixed-methods study with a one-group pretest and posttest design in a particular geographic target zone (approximately 22-mile diameter) within a much larger metropolitan area of Southern California. Purposive sampling methods were used for community-based recruitment from May 2015 to August 2015. The research was designed so that participants could complete all aspects of the study entirely over the phone and Internet.

### Participants

Inclusion criteria limited enrollment to those who self-identified as Latina women aged 21 to 55 years who read and spoke English and who scored above the threshold at screening for depression on the Patient Health Questionnaire (PHQ-9) [41] or the brief 7-item Generalized Anxiety Disorder scale (GAD-7) [42]. Additional inclusion criteria required participants to have access to the Internet via a smartphone, tablet, or computer. The study flier clarified that each step of the study would be done over the phone and the Internet using a smartphone, tablet, or computer and that it involved watching some videos including a television-like story featuring a Latina character.

A total of 63 women called the study phone. Of the 63 callers, 28 were ineligible based on the inclusion criteria, including 6 who expressed suicidal ideation (they were further screened and referred using a suicide protocol), 10 who scored below threshold for both depression and anxiety, 4 who did not speak English, 2 who did not identify as Latinas, 4 who did not complete the screening, and 2 who reported they had only called to inquire for a friend. Thus, 35 women moved on to be screened and met eligibility requirements. Of them, 4 did not complete the Web-based informed consent after screening, 2 consented but did not participate beyond giving consent, 1 consented but later contacted the research assistant (RA) to report she could not gain access to the Internet on her phone. A final total of 28 eligible women moved beyond consent, and all of them completed the study; that is, there was no attrition over the 6 weeks of data collection.

### Procedure

**Figure 1:**
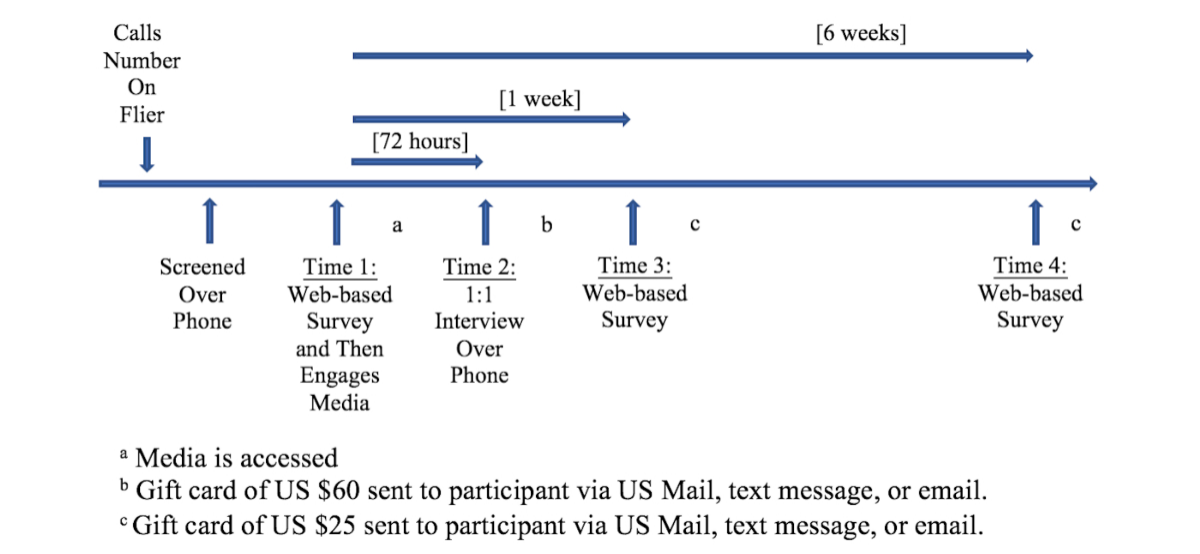
Timeline of data collection and gift card distribution time points.

Fliers were distributed at nine partner sites including women’s health clinics, parenting centers, social service sites, and a large catholic church in the geographic target zone. The fliers, printed in color and featuring a photo of the Latina main character from the story, invited adult Latinas who were struggling with feelings of sadness or worry to call if they wanted to participate in a media-based study. After giving verbal consent, callers were screened by the RA, a psychiatric mental health nurse practitioner (second author). The RA administered the PHQ-9 [41] and the GAD-7 [42] at screening, documenting all answers and scoring in real time. Subsequently, the data were entered into Statistical Package for the Social Sciences (SPSS, IBM Corp) for comparison to posttest answers. An IRB-approved protocol was followed for women who self-identified as having suicidal ideation; they were not enrolled. See Figure 1 for the intervention protocol beginning with screening and moving from Web-based informed consent, to media engagement, and online surveys.

**Figure 2:**
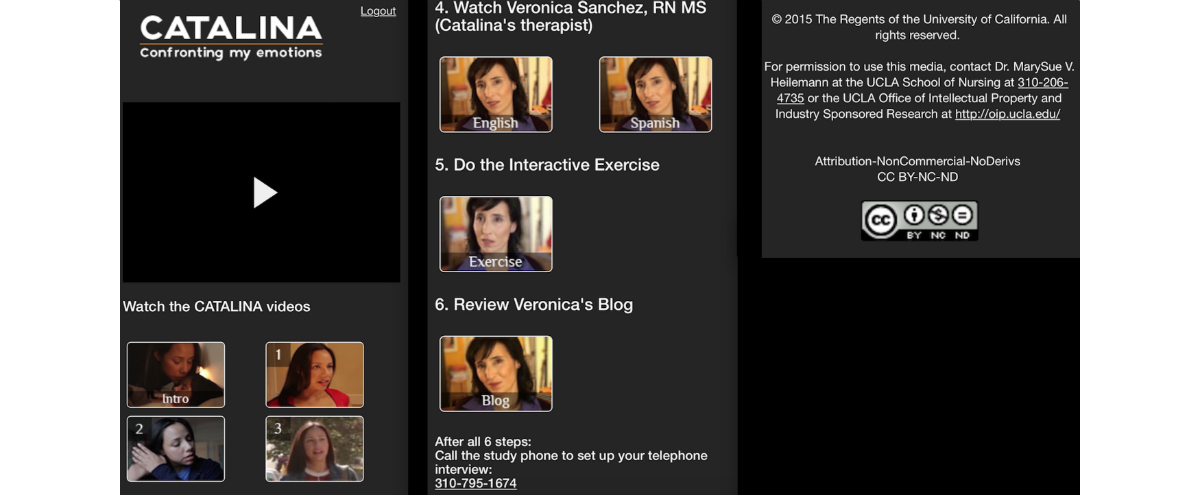
Screenshots of smartphone view (3 of 3 screenshots).

All enrolled participants were assigned a confidential identification number for deidentification purposes, and media was made accessible via a password protected “Catalina” website after they completed the Web-based informed consent using a link sent to them in a text message (short message service, SMS) or email. Participants first completed at time 1 the online survey and then accessed the website that included an introductory video along with a series of links that were sequentially arranged to guide the viewer. Figure 2 displays screenshots of the smartphone view, including how the videos were numbered. The introduction video led to a 3-min overview video introducing the project. The first link after the “intro” led to a 14-min video that depicted a story featuring the main character named Catalina, a 28-year old Latina who was dealing with life challenges including symptoms of depression and anxiety in her daily life at home, at a party, and alone with a friend. The second and third links led to two shorter 4-min bonus videos that extended the story; one portrayed Catalina grappling with the decision to seek therapy, and the other showed her reflecting on her experience after having had a positive therapy session with a Latina nurse-therapist character whom we named Veronica Sanchez, RN. The fourth link led to a bonus video of Veronica alone speaking directly to the viewer as a Latina, a nurse, and a therapist and providing user-friendly psychoeducation about depression and anxiety, as well as information on treatment and help-seeking. The Latina character of Veronica was portrayed as a nurse who was a therapist because of the relatively high trust that the public [43] and Latinos have in nurses [44,45], including nurses as therapists who treat depression [21], to counteract the stigma associated with mental health care [13]. The bilingual actress who played Veronica did both English and Spanish versions of one specific psychoeducational bonus video so that participants could share the Spanish version with friends or family who did not speak English.

**Figure 3:**
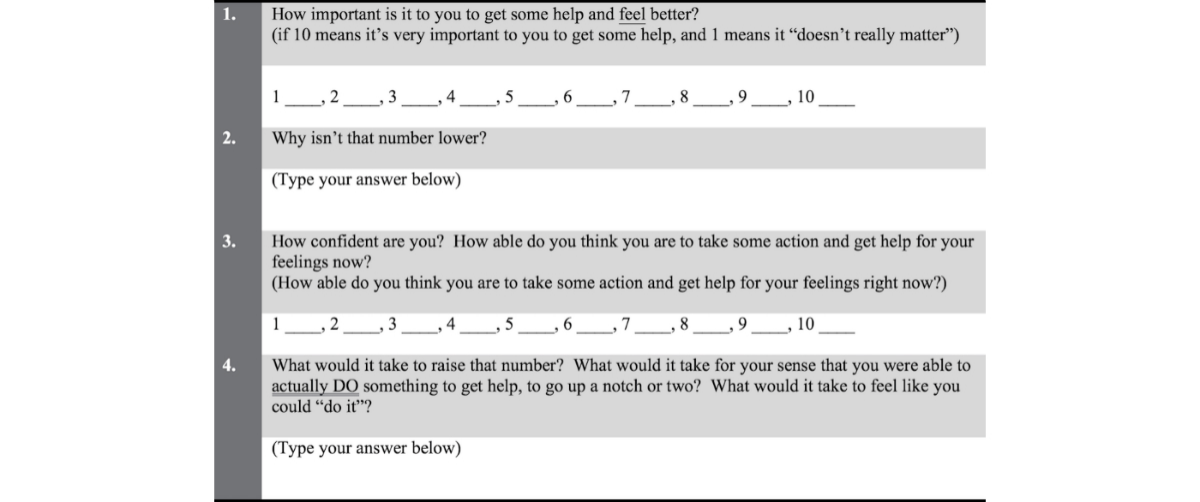
Web-based questions for interactive video sequence based on Rollnick and Miller’s (2008) motivational interviewing ruler.

Link number five led to a series of coordinated short video segments wherein Veronica (the nurse-therapist character) directly asked the viewer specific questions inspired by motivational interviewing (MI) [46] in a sequence lasting about 6 min in total to spur participants’ thinking about themselves and their situation. For this interactive sequence, the viewer answered by choosing or clicking on a number and also by typing words into textboxes (see Figure 3 for the script of Web-based questions that women answered) and then clicking to go to the next video in the sequence. The aim of the successive videos and questions, controlled by the viewer, was to allow participants to move at their own pace while reflecting on how they were feeling or thinking [47]. At the end of the sequence, Veronica provided psychoeducation and explained how women could use their answers to reflect on their intentions. She then invited women to visit a blog that was accessible via the final (sixth) link; the blog included textual content written from Veronica’s point of view and featured names, addresses, and Internet links to various affordable mental health resources for individuals with low incomes, including local clinics and hotlines.

At all points, viewers were free to decide if and when they would click any links to engage with the surveys or media. The Web page included a reminder for participants to call the study phone after engaging with the media to schedule a one-to-one interview with the RA within 72 hours of media exposure. The qualitative telephone interview explored women’s perceptions of the media in a way that protected women’s privacy. As reported elsewhere (in process), the individual interviews were audiorecorded, transcribed, and analyzed. Participants were sent a text message 1 and 6 weeks after engaging in the media, which provided a link to Web-based surveys (time 3 and time 4) that included the PHQ-8 and GAD-7 plus survey questions about perceptions related to the importance of getting immediate help, confidence they could get help, and intentions or action taken. To compensate for their time, gift cards to a local retailer were mailed, emailed, or texted to participants at three points (see Figure 1).

### Measurements and Instruments

As few studies have examined the integration of health information into transmedia storylines, Bowen’s [48] standard areas of feasibility were used to guide these analyses. Thus, our analyses included the criteria of: implementation, demand, acceptability, and limited efficacy. Implementation addressed ease of access and degree of engagement of the delivery of the intervention (how many women accessed the Web page, navigated the media site, and answered MI interactive questions by typing within answer boxes without needing assistance). Demand involved the extent to which the transmedia intervention was used (how many women watched and completed all minutes of each video). Acceptability involved suitability, attractiveness, or satisfaction of the intervention (answers to questions about relatability to the main character and desirability of seeing the nurse-therapist character and how many women used or shared Veronica’s blog). Limited efficacy involved the preliminary effectiveness of the transmedia intervention to reduce symptoms of depression and anxiety and to influence perceived importance of getting help and perceived confidence that they could seek help.

#### Depressive Symptoms

The PHQ-9 [49] is a 9-item questionnaire for depression and was used at the time of initial screening to measure the severity of depression, including suicidal ideation experienced during the preceding 2 weeks. However, the PHQ-8 was used for two subsequent time points of the study without the ninth item about suicidal ideation (time 3 and time 4). Response options for the PHQ-9 and PHQ-8 are on a 4-point Likert scale and range from “not at all” to “nearly every day.” PHQ-9 scores indicating either moderate (≥10) or severe (≥15) depression represented study eligibility. The PHQ-9 for depression had good internal consistency reliability (Cronbach alpha=.86) [41] and good criterion validity (*r*=.79) in a sample of 3000 women from obstetrician or gynecologist clinics. In another study with 6000 patients, the PHQ-9 demonstrated good sensitivity (88.0%) and specificity (88.0%) to discriminate between depressed and nondepressed individuals at the cut-point of 10 or higher [49]. Merz and colleagues [50] did a study with a community-based sample of 479 Latinas and found the Cronbach alpha for the English version of the PHQ-9 to be .84 and for the Spanish version to be .85. Any response other than *not at all* for the suicidal ideation item on the scale indicated possible suicidal ideation [51], and therefore, for this study, a suicide risk assessment protocol was implemented if any woman endorsed this item.

After screening, the PHQ-8 was used to measure the severity of depression over the past 2 weeks [52]. For this study, the PHQ-8 was completed 1 and 6 weeks after engaging with the transmedia (time 3 and 4, respectively). Questionnaire items for the PHQ-8 are identical to those of the PHQ-9 except for the omission of the suicidal ideation item. Reliability was high in a sample of 32 Bolivian primary care patients (78%, 25/32 women) who were assessed by an automated telephonic depression self-care service using the PHQ-8 with a Cronbach alpha of .83 [53]. In a sample of 1022 coronary artery disease patients, the sensitivity and specificity for detecting major depression were similar for both the PHQ-9 (54% and 90%, respectively) and PHQ-8 (50% and 91%, respectively) [51]. Furthermore, a high correlation between the PHQ-9 and PHQ-8 scores (*r*=.997) was found [51].

#### Anxiety Symptoms

The GAD-7 is a 7-item questionnaire for anxiety and was used to screen for and measure the severity of anxiety over the preceding 2 weeks in this study [54]. Response options are on a 4-point Likert scale and range from *not at all* to *nearly every day*. GAD-7 scores indicating either moderate (≥10) or severe (≥15) anxiety represented study eligibility. Anxiety severity was assessed at screening and 1 and 6 weeks after engaging the transmedia intervention (time 3 and 4, respectively). In a study by Mills and colleagues [54] with a community sample of 436 Latinos, the GAD-7 had strong internal consistency reliability with a Cronbach alpha of .91 for the English version and .94 for the Spanish version.

#### Levels of Perceived Confidence and Importance

For the interactive video sequence with Veronica, questions were fashioned after Rollnick and Miller’s [47] MI ruler and were created to capture motivational dimensions for help-seeking behavior (see Figure 3). In one of the videos in the interactive sequence, a single-item question was used to measure women’s perceptions of how important it was to get emotional help right now. Possible responses ranged from 1 to 10 with 1 meaning it was not very important and 10 meaning it was very important. Another single-item question with a 10-point range was used in the interactive sequence to measure women’s perceptions of how confident they were in their ability to actually do something to get emotional support at this time (1 indicated they were not confident and 10 indicated they were very confident). Higher scores represented greater importance and greater confidence, respectively.

#### Behavior Change

Action taken and intentions to take action to get mental health care or support were assessed using Web-based surveys at time 3 and time 4 (see Figure 1), with a single question that had four possible answers. The answers ranged from (1) denial of action taken and no intention to act, to (2) denial of action but actively considering taking action to get help, to (3) denial of action but having certainty of intention to act in the future, to (4) affirmation that action had been taken to get support.

#### Relatability and Desirability

Included in the time 2 telephone interview (scheduled after engaging the transmedia intervention) was a single-item Likert question with a 10-point range that was used to measure how much the women related to the main character (Catalina). Possible responses ranged from 1 to 10, with 1 meaning they did not relate to her at all and 10 meaning they completely related to her. Another single-item Likert question was asked to measure how comfortable women would be seeing someone such as the nurse-therapist character (Veronica) for counseling (1 indicated there were not comfortable and 10 indicated they were very comfortable).

#### Impact of Transmedia

Women were asked if they thought about or talked about the videos with family members, friends, or anyone else since viewing the media. These questions were asked as part of the Web-based surveys 1 and 6 weeks after engaging with the media (time 3 and 4, respectively; see Figure 1). Possible answers included (1) No, (2) Yes, and (3) Choose not to answer.

#### Use of Blog

Participants were asked if and how they used Veronica’s resource-rich blog about where to seek services for support or therapy. Actions taken and intentions to act were measured as part of the Web-based surveys 1 and 6 weeks after engaging with the media (time 3 and 4, respectively; see Figure 1). Possible answers were (1) No, (2) Yes, and (3) Choose not to answer. Another single-item question using the same answer choices was used to measure if women shared resources found on Veronica’s blog with family, friends, or anyone in need at time 3 and time 4 (see Figure 1).

#### Access to and Engagement With Intervention Content

Web-based analytics tracking was used to measure the number of women who, without contacting the RA for assistance, (1) accessed the transmedia intervention Web page, (2) accessed and watched all videos, (3) completed all minutes of each video, and (4) answered the Web-based MI interactive questions.

### Data Analysis

Descriptive statistics were computed for sample characteristics. To assess feasibility in terms of implementation, descriptive statistics were used to compute how many women accessed, engaged, and navigated the Web page and answered the MI interactive questions (by typing within answer boxes) without assistance. To assess demand, descriptive statistics were used to compute how many women watched and completed all minutes of each video. To assess acceptability, descriptive statistics were used to compute relatability to the main character, desirability of seeing the nurse-therapist character, and the number of women who used or shared information on Veronica’s blog. Limited efficacy was assessed using repeated measures analysis of variance to assess change over time for anxiety and depression symptoms and Spearman correlations to evaluate the association of perceived confidence and importance of getting immediate help with anxiety, depression, and action taken.

## Results

### Sample

Of the 28 participants who completed the Web-based consent form and accessed the media Web page (see Figure 1), 23 women (82%, 23/28) completed the telephone interview within 72 hours of viewing the transmedia, 4 (14%, 4/28) completed the interview within 6 days, but 1 completed the interview after 14 days. The average interview length was 45 min for each participant (range: 29-75 min). Participants were asked to complete surveys 1 and 6 weeks after viewing the transmedia (time 3 and 4, respectively). The average time between viewing and completion of the time 3 survey was 7.5 days (range: 6-16 days). The average time between viewing and the time 4 survey was 6 weeks (range: 6-7.5 weeks).

See Table 1 for demographic information of the sample. The mean age was 29.2 (standard deviation 7.1.). More than half of the sample finished high school and attended some level of post high school education such as college or technical school. Most participants reported they found it difficult or very difficult to pay their weekly bills.

In terms of TV viewing habits, most participants reported that they watched TV shows about characters in a comedy or drama on a weekly basis. Over half of the sample watched story-based dramas or comedies several times weekly, and over three-quarters of the sample watched telenovelas on a weekly basis. In terms of the Internet, all participants reported that they watched videos, movies, or story-based shows via a smartphone, tablet, or computer on a weekly basis (see Table 2).

### Intervention Feasibility

As noted above (see Participants), 1 woman enrolled in the study but did not have Internet access on her phone so did not stay in the study. Of the 28 women who enrolled and had access to the Internet, all engaged the media using a smartphone, tablet or computer, and all stayed in the study throughout the 6 weeks.

#### Implementation

In terms of Bowen’s feasibility criteria of implementation [48], all 28 women clicked the media link to engage with the videos without assistance from the RA. Likewise, all 28 typed in answers to the MI interactive questions without need for assistance.

#### Demand

The website tracker of user plays for each video showed that all 28 women in the sample watched all the videos. Of the total, 27 women completed all minutes of all videos; however, 1 woman stopped the 14-min video approximately 2.5 min before its ending.

#### Acceptability

The mean relatability score for how much women related to the main character (Catalina) was 6.95 on a 10-point scale. The mean desirability score for how comfortable women would be seeing the nurse-therapist character for counseling (Veronica) was 8.13 on a 10-point scale. Within 6 weeks of viewing, 20 of the 28 women (71%, 20/28) used or shared with others the blog or information from the blog.

### Limited Efficacy Analysis

#### Depressive Symptoms

**Figure 4:**
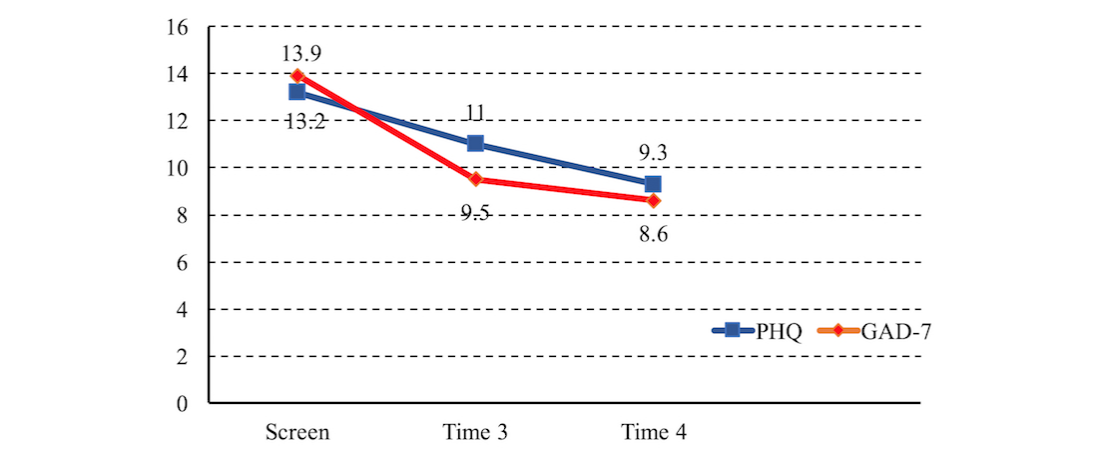
Mean depression (Patient Health Questionnaire 9-item, PHQ-9 or 8) and anxiety (Generalized Anxiety Disorder scale 7-item, GAD-7) scores at screening, 1 week (time 3) and 6 weeks (time 4) after engagement with the transmedia intervention (PHQ-9 at screening; PHQ-8 at time 3 and time 4).

Depressive symptom levels decreased significantly across time (*F*_2,54_=9.0, *P*<.001); there was a linear decrease (*F*_1,27_=14.2, *P*=.001) with scores at screening significantly higher than at 1 week (*F*_1,27_=6.45, *P*=.02) and 6 weeks after exposure to the media (*F*_1,27_=14.2, *P*=.001; see Table 3). Even though results showed a continuing decrease across time, there was a slight lessening of the decrease from week 1 to week 6 after media exposure such that the 1- and 6-week scores were not significantly different (*F*_1,27_=3.9, *P*=.06; see Table 3 and Figure 4).

#### Anxiety Symptoms

For anxiety symptoms, there was a significant reduction across time (*F*_2,54_=18.7, *P*<.001). A quadratic function also describes the pattern (*P*=.008) showing a “curve,” with greater decrease between screening and 1 week post media than from 1 to 6 weeks after media exposure (see Table 3). Scores at screening were significantly higher than at 1 week post media exposure (*F*_1,17_=25.5, *P*<.001) and at 6 weeks post media exposure (*F*_1,27_=23, *P*<.001). Even though results showed a continuing decrease across time, there was a lessening of the decrease from week 1 to week 6 post media exposure such that the week 1 and week 6 scores were not significantly different (*F*_1,27_=1.4, *P*=.25; see Table 3 and Figure 4).

**Table 1:** Sample demographics (N=28).

Demographic variables			n (%)
**Education**			
	**Highest education level**		
		Some high school (but not all)	3 (10.7)
		Graduated from high school or earned a general educational development certificate	7 (25)
		Some technical, trade, or vocational school after high school	3 (10.7)
		Attended some college	8 (28.6)
		Graduated with an associate degree	1 (3.6)
		Graduated with a bachelor’s degree	3 (10.7)
		Graduated with a master’s degree	2 (7.1)
		Chose not to answer the question	1 (3.6)
**Finances**			
	**Ability to meet weekly financial needs**		
		Not difficult	3 (10.7)
		Somewhat difficult	6 (21.4)
		Very difficult	19 (67.9)
		Choose not to answer	
			
**Mental health symptoms**			
	**Depression and anxiety**		
		Depression: PHQ-9^a^ score ≥10	3 (10.7)
		Anxiety: GAD-7^b^ score ≥10	4 (14.3)
		Depression and anxiety scores each ≥10	21 (75)

^a^PHQ-9: Patient Health Questionnaire 9-item.

^b^GAD-7: Generalized Anxiety Disorder scale 7-item.

**Table 2:** Media viewing habits of sample using television (TV) and Internet (N=28).

Media viewing habits		n (%)
**How often do you watch TV shows that are stories about characters in a situation comedy or drama?**		
	Never	1 (3.6)
	Once a week	5 (17.9)
	Several times a week	15 (53.6)
	Everyday	7 (25)
**How often do you watch telenovelas on TV?**		
	Never	6 (21.4)
	Once a week	5 (17.9)
	Several times a week	13 (46.4)
	Everyday	4 (14.3)
**How often do you watch videos from the Internet on your smartphone, tablet, or computer?**		
	Never	0
	Once a week	7 (25)
	Several times a week	12 (42.9)
	Everyday	9 (32.1)
**How often do you watch movies or story-based shows on your smartphone, tablet, or computer?**		
	Never	5 (17.9)
	Once a week	8 (28.6)
	Several times a week	9 (35.7)
	Everyday	5 (17.9)

**Table 3:** Mean scores (standard deviation) for depression and anxiety (n=28) 1 and 6 weeks after engagement with transmedia intervention.

Mental health scores	Screening Mean (SD^a^)	1 week after media (time 3) Mean (SD)	6 weeks after media (time 4) Mean (SD)
Depression PHQ-9^b^	13.2 (3.9)	-	-
Depression PHQ-8^c^	-	11.0 (5.6)	9.3 (6.7)
Anxiety GAD-7^d^	13.9 (3.4)	9.5 (4.6)	8.6 (6.1)

^a^SD: standard deviation.

^b^PHQ-9: Patient Health Questionnaire 9-item.

^c^PHQ-8: Patient Health Questionnaire 8-item

^d^GAD-7: Generalized Anxiety Disorder scale 7-item.

#### Perceived Confidence and Symptoms

Six weeks after media exposure, symptoms of depression and anxiety were both significantly correlated with perceived confidence that participants could take action to get immediate help. That is, higher levels of confidence were associated with lower levels of depression symptoms (Spearman ρ (rho): −.399, *P*=.04) and with lower levels of anxiety symptoms (Spearman ρ: −.460, *P*=.01).

#### Perceived Importance and Symptoms

Six weeks after media exposure, women’s perceived level of importance of getting help for their emotions was not significantly correlated with anxiety symptoms (Spearman ρ: −.288, *P*=.14), nor was it significantly correlated with depression (Spearman ρ: −.258, *P*=.19). These moderate but nonsignificant effects indicated that higher perceived levels of importance were associated with lower levels of depression and anxiety.

#### Intentions or Actions Taken to Seek Help

Within 1 week of media exposure, participants had changed their behavior. Specifically, 11 women (39%) reported they had already used the resources from the blog including links and contact information of local resources for support or therapy and hotline numbers. In addition, within 1 week, 25 women (89%, 25/28 of the sample) reported thinking about the videos during the week, and 23 women (82%, 23/28) reported they had already talked about the videos and transmedia with friends or family. Six weeks after exposure to the media, 8 women (28.5%, 8/28) reported they had used resources from the blog, and 21 women (75%, 21/28) reported they were still spending time thinking about the videos. At time 4, a total of 22 women (79%, 22/28) reported they had talked about the videos since time 3 when they completed the last set of Web-based questions 5 weeks earlier.

#### Perceived Confidence and Intentions or Actions Taken to Seek Help

Women’s level of perceived confidence in their ability to seek help was significantly associated with intentions or taking action to get help both 1 week after (Spearman ρ: .513, *P*=.005) and 6 weeks after engaging with the media (Spearman ρ: .410, *P*=.04). Higher levels of confidence were associated with higher levels of intentions or taking action to get help both 1 week and 6 weeks after engaging with the media.

#### Perceived Importance and Intentions or Action Taken to Seek Help

Women’s perceptions of the importance of getting help for their emotions was significantly associated with intentions or taking action to get help both 1 week after (Spearman ρ: .487, *P*=.009) and 6 weeks after engaging with the media (Spearman ρ: .554, *P*=.003). Higher levels of importance were associated with higher levels of intentions or taking action to get help both 1 and 6 weeks after engaging with the media.

## Discussion

### Principal Findings

Preliminary findings indicate that an Internet-accessible transmedia storytelling intervention is a feasible approach for engaging and helping English-speaking Latina adults with symptoms of depression, anxiety, or both. Such interventions hold promise for reaching much larger numbers of Latinas, including those who are underserved but need help. To our knowledge, this is the first mental health transmedia intervention, and findings suggest that women found the intervention compelling, therapeutic, and resourceful. Participants engaged with all features of the intervention, and all of them remained in the study for the entire 6-week duration; there was no attrition after initiation of the study.

Importantly, women in our sample took action, and within 1 week, more than a third (39%, 11/28) used resources from the resource-rich blog. This included referral sites and hotlines that women used to gain knowledge about the available resources, to gather information, or to make an appointment for services. Moreover, the participants found the transmedia to be socially valuable, with the majority (82%, 23/28) sharing it with those in their social circle within days of viewing it for the first time. Rather than keeping their experience of the transmedia to themselves, most of the women in our sample told friends and family about the transmedia intervention without delay. These findings highlight the convenient and strategic design of the transmedia website. Participants who determined that they (or a friend) could benefit from services just as Catalina did, could readily access resources.

Finally, the intervention was therapeutic in that symptoms reduced after engagement with our transmedia storytelling intervention. Our 6-week intervention led to a statistically significant improvement of the debilitating symptoms of depression and anxiety. This drop in symptom levels over the 6-week intervention illustrates how a digital storytelling intervention using the Internet can make a measurable difference in an individual’s life. This is even more important because of our sample’s vulnerability; 75% (21/28) of our Latina participants reported co-occurring depression and anxiety, which is commensurate with the literature [3]. Co-occurrence rates are estimated to be as high as 50% [55], which is associated with greater chronicity, psychiatric hospitalization, psychosocial disability, and suicide [56]. The reduction of symptoms and improvement in confidence for seeking mental health services was possible in just 1 week with the Catalina intervention, and symptoms further reduced through the 6-week point. This implies that further reduction of symptoms and further help seeking action may be possible if more story-based episodes, psychoeducational bonus videos, or media-based interactive exercises would be made available on an ongoing basis.

Compelling, story-based content that individuals find appropriate, desirable, and easily accessible via the Internet provides a way to reach thousands of people about topics that are often considered taboo. For example, the *East Los High* resource Web page associated with the show provided a hyperlink to Planned Parenthood, which was used 26,414 times during the first season and another 30,868 times in the 6 weeks after the season ended [26]. As mental health–related stigma within the Latino community has led to concealment of potential mental health problems, avoidance of seeking needed mental health services has been problematic [57,58]. Internet-based transmedia, however, offers a way to do discreet outreach to help symptomatic women overcome stigma-related barriers through a story that women find interesting. By considering how a fictitious character is dealing with their emotional issues, women can reflect in a low risk way on how they are feeling and consider ways to get help. Due to the individual nature of mental health challenges, our transmedia helped optimize privacy in that women used their own personal devices to access and explore the media at their own discretion without others having to know what they were doing. Women had control over when, where, and how long they engaged the media, which maximized their ability to use it at a time convenient for them. Overall, our findings indicate that the women were receptive to a Web-based psychotherapeutic intervention.

It is key for story-based edutainment to have realistic stories set in a believable world that is socioculturally relevant with a main character that viewers can relate to [59,60] and identify with [31]. We drew upon previous data, including findings from English-speaking Latinas treated for depression [18,20,21] to create the main character (Catalina) and the nurse-therapist character (Veronica). Likewise, the script for our intervention was based on deidentified data about real-life experiences of Latinas struggling with their emotions. It was edited on the basis of feedback from a team of therapists; subsequently, it was theater-tested with a community sample of Latina adults (manuscript in process) and edited again. These methodological steps were valuable in that results showed participants demonstrated strong character identification with the main character (Catalina). Furthermore, participants found the nurse-therapist character (Veronica) to be highly acceptable as someone they would be comfortable seeing for mental health care. These scores suggest that participants shared certain key characteristics with Catalina and that they were attracted to the character of Veronica, portrayed as a Latina woman who was both a nurse and a therapist, in relation to an imagined working relationship [61]. Within the story, Catalina referred to Veronica as her therapist whom she saw as a capable health professional she could trust. Veronica was featured providing psychoeducational content, leading the interactive sequence using Rollnick and Miller’s [46] MI ruler, offering participants guidance on how to interpret their answers, and inviting women to use her resource-rich blog. It is possible that the effectiveness of our transmedia intervention was enhanced because of the rapport women experienced with Veronica. It is likely she was perceived by participants not only as Catalina’s confidante but potentially their own as well. As nurses are viewed as the most honest and ethical professionals [43] and as evidenced from previous research, a nurse who has specialized training as a therapist has been effective with depressed Latinas [21].

In conjunction with symptom reduction, transmedia storytelling interventions may also serve as catalysts that help women take action to seek mental health services. For our sample, both perceived confidence (self-efficacy) and importance were associated with increased actions taken to get help 1 and 6 weeks after viewing the media. On the basis of Bandura’s social cognitive theory, self-efficacy is a psychological determinant of behavior change commonly targeted in health promotion interventions [62]. Other research on the ability to perform mental health care activities using the mental health self-efficacy scale offers support for linking confidence, self-efficacy, media, and mental health symptoms; Clarke and colleagues [63] studied a sample of adults suffering from mild-to-moderate symptoms of depression, anxiety, and stress and found that a Web-based psychotherapeutic intervention was associated with increased confidence for managing common mental health problems, along with a reduction in depression, anxiety, and stress [63]. Thus, if Catalina, who struggled similarly with life’s daily obstacles and emotions in the story was able to find help, participants may also have felt confident in their ability to take risks to explore Web pages, make calls or appointments, and seek help.

### Limitations

This was a feasibility study with a one-group pretest posttest design. Thus, results cannot be generalized beyond this sample until an attention control and usual care are added to the research, as necessary, to verify the efficacy for this transmedia intervention. With the surveys and the qualitative interview administered by the RA, a psychiatric mental health nurse practitioner, the possibility of a therapeutic effect exists.

### Conclusions

Because telenovelas have long been enjoyed by Latina audiences and because of the success of *East Los High* [26], compelling transmedia dramas hold promise for reaching many Latinas who are at risk themselves or who know of someone who is. With the potential to reach thousands of women through Web-based content, the impact on women’s knowledge of symptom management options could be sizable. The results of our study support the hypothesis that a transmedia intervention, informed by deidentified data composites from at-risk Latinas, can be developed and is able to affect symptoms and spur action. In addition to a randomized controlled trial, what remains to be studied are the enduring effects of the intervention such that participants continue to display enough confidence to seek and obtain the therapy they need.

Future implications of this study include advancements in the use of transmedia, such as further development of more interactive Web-based activities to access other evidence-based therapeutic help for women to make strides in managing their symptoms. This research and development may provide insight into how to refine and tailor future evidence-based Web-based treatment programs for Latinas. A recent review of Internet-based cognitive behavioral therapy (iCBT) supports the efficacy of iCBT to successfully treat depression and anxiety and particularly in overcoming the barriers that often preclude treatment [64]. Due to the privacy possible when engaging an intervention such as ours using transmedia via one’s own personal smartphone, tablet or computer, such interventions may help women overcome stigma, increase confidence to seek care, or expand options for helpful resources to help them tolerate long wait-lists encountered when seeking needed therapy.

## Abbreviations

GAD-7: Generalized Anxiety Disorder scale 7-item
iCBT: Internet-based cognitive behavioral therapy
MI: motivational interviewing
PHQ-9: Patient Health Questionnaire 9-item
RA: research assistant
SD: standard deviation
UCLA IRB: University of California, Los Angeles Institutional Review Board
